# New specific skeletal muscle mass index cut-offs for the assessment of sarcopenia in patients with severe obesity

**DOI:** 10.3389/fendo.2024.1369584

**Published:** 2024-07-05

**Authors:** Annalisa Bufano, Alessandra Cartocci, Nicoletta Benenati, Cristina Ciuoli, Maria Simon Batzibal, Alessio Bombardieri, Gabriele Iraci Sareri, Ida Sannino, Andrea Tirone, Costantino Voglino, Giuseppe Vuolo, Maria Grazia Castagna

**Affiliations:** ^1^ Department of Medicine, Surgery and Neuroscience, Unità Operativa Complessa (UOC) Endocrinology, University of Siena, Siena, Italy; ^2^ Department of Medical Biotechnologies, University of Siena, Siena, Italy; ^3^ Department of Surgical Sciences, Bariatric Surgery Unit, University of Siena, Siena, Italy

**Keywords:** obesity, sarcopenia, muscle, SMI, BIA

## Abstract

**Introduction:**

Bioelectrical impedance analysis (BIA) is the most used tool in clinical practice to evaluate body composition in patients with obesity. The skeletal muscle index (SMI) defined by BIA has been proposed for the identification of sarcopenia, but there are currently no univocal cutoffs for this condition. In this study, we aimed: 1) to determine the prevalence of sarcopenia in patients with severe obesity using the current cutoffs of SMI; 2) to define new specific cutoffs; 3) to validate the new cutoffs; and 4) to re-determine the prevalence of sarcopenia.

**Methods:**

A total of 300 patients, 74% women and 26% men (mean age = 42.6 ±; 9 years), with morbid obesity (mean BMI = 46.7 ±; 6.5 kg/m^2^) followed by the Unit of Endocrinology from January 2014 to December 2020 were retrospectively evaluated. SMI was calculated as the skeletal muscle mass normalized for squared height through the BIA equation by Janssen et al.

**Results:**

The prevalence of sarcopenic obesity calculated using the cutoff points reported by De Rosa et al. (7.3 kg/h^2^ for women and 9.5 kg/h^2^ for men) was 2.3%. The prevalence of sarcopenia was calculated using the new cutoffs: with the cutoff obtained from the standard deviation method (8.2 kg/h^2^ for women and 10.2 kg/h^2^ for men), a prevalence of 14.7% was observed, whereas the prevalence reached 47.6% when using the cutoff calculated through the *K*-means unsupervised cluster (9.2 kg/h^2^ for women and 11.3 kg/h^2^ for men). The new cutoffs were validated with a second sample consisting of 300 patients with morbid obesity (BMI = 44.9 ±; 6.7 kg/m^2^): the rate of sarcopenic patients was still higher than that observed in the training cohort (56%). After the matching procedure (by BMI and age), the rates of sarcopenic patients were similar in both groups (50.2% in the validation group and 53% in the training group, p *= 0.6).*

**Conclusion:**

The new cutoffs calculated with cluster analysis could better identify sarcopenia in morbidly obese patients. However, further studies are needed to validate these cutoffs in different patient cohorts.

## Introduction

The term “sarcopenic obesity” (SO) has been proposed to identify the coexistence of obesity and sarcopenia (1). Sarcopenia, which is characterized by low skeletal muscle mass (SMM) and function, is largely present in the geriatric population, with its prevalence increasing with age (2–4). Nevertheless, sarcopenia can also be present in patients with obesity and associated comorbidities (e.g., heart failure and endocrinological and metabolic diseases) (5). Independently of age, obesity can lead to loss of muscle mass and function due to the negative impacts of adipose tissue-dependent metabolic disorders, such as oxidative stress, inflammation, and insulin resistance, all of which negatively affect muscle mass (6).

There are different diagnostic tools useful for the evaluation of muscle mass, such as computed tomography (CT), magnetic resonance imaging (MRI), dual-energy X-ray absorptiometry (DXA), and bioelectrical impedance analysis (BIA). The use of MRI for evaluation is considered the gold standard; however, the costs, the risks related to radiation exposure, and the weight of patient have limited its use in patients with obesity. In contrast, BIA is easy to use and has low costs, wide availability, and portability. It is therefore the most used tool in clinical practice for patients with obesity (3). However, BIA is influenced by the patient’s hydration status (7), and unlike MRI and CT, it is not able to evaluate intramuscular fat (8).

In 2000, Janssen et al., from assessments of body composition through BIA, proposed the SMM index (SMI) for the definition of sarcopenia (9).

The European Society for Clinical Nutrition and Metabolism (ESPEN) and the European Association for the study of Obesity (EASO) believe that greater attention needs to be paid to the prevention and development of strategies for the treatment of this clinical state and, recently, have proceeded to publish a consensus to establish a definition and univocal diagnostic criteria (10). According to this consensus, the diagnosis of SO will be performed in two steps: firstly, with the assessment of skeletal muscle functional parameters; subsequently, the diagnostic algorithm will continue with the assessment of body composition using DXA, BIA or, when possible, CT (10).

However, there is currently no univocal cutoff for SMI assessed by BIA that is valid for the definition of sarcopenia in patients with morbid obesity. Therefore, the real prevalence of sarcopenia is not known, but ranges from 4.4% to 84.0% in men and from 3.6% to 94.0% in women (11). A recent systematic review has highlighted the great heterogeneity of the data present in the literature in terms of the diagnostic criteria and the SMI cutoffs, which does not allow firm conclusions (12).

The aims of this study were: 1) to determine the prevalence of sarcopenia in patients with severe obesity using one of the current cutoffs of SMI as defined by BIA; 2) to define new specific cutoffs of the SMI in patients with severe obesity; 3) to validate the new cutoffs; and 4) to re-determine the prevalence of sarcopenia using the new cutoffs in a validation cohort of patients with severe obesity.

## Materials and methods

### Study population

The baseline anthropometric characteristics of the whole cohort and according to gender are summarized in **Table 1**. A total of 300 patients, 222 women (74%) and 78 men (26%), with mean age of 42.7 ±; 9.1 years, with severe obesity (mean BMI = 46.7 kg/m^2^ ±; 6.5) were retrospectively evaluated. The patients were followed by the Unit of Endocrinology from January 2014 to December 2020, and they were candidates for bariatric surgery (Roux-en-Y gastric bypass or one anastomosis gastric bypass) performed at the Bariatric Surgery Unit. The inclusion/exclusion criteria are those for bariatric surgery according to the SICOB (Italian Society of Obesity Surgery) guidelines (https://www.sicob.org/00_materiali/Linee_Guida_SICOB_2023.pdf):

- Age between 18 and 65 years;
- Body mass index (BMI) ≥40 kg/m^2^;
- BMI ≥35–39.9 kg/m^2^ with at least one associated comorbidity (e.g., diabetes, hypertension, dyslipidemia, or obstructive sleep apnea syndrome); and
- BMI of 30–34.9 kg/m^2^ and at least one comorbidity with poor control despite medical therapy.

**Table 1:** Anthropometric features and body composition of the whole cohort and according to gender (training group).

Parameters	All (*N* = 300) Mean ± SD	Men (78, 26%) Mean ± SD	Women (222, 74%) Mean ± SD
**Age (years)**	42.7 ±; 9.1	43.3 ±; 9.8	42.5 ±; 8.8
**Weight (kg)**	127 ±; 21.6	142.4 ±; 22.5	121.5 ±; 18.5
**BMI (kg/m^2^)**	46.7 ±; 6.5	47 ±; 6.3	46.6 ±; 6.6
**EW (kg)**	41.5 ±; 10.7	43.7 ±; 12	40.7 ±; 10.1
**WC (cm)**	128.7 ±; 14.9	138 ±; 11.7	125.4 ±; 14.5
**HC (cm)**	133.3 ±; 13	130.8 ±; 12.5	134.1 ±; 13
**R (Ohm)**	462.9 ±; 68.3	417.6 ±; 68.4	478.8 ±; 60.9
**FFM (kg)**	60.4 ±; 14	75.7 ±; 15.8	55 ±; 8.1
**FM (kg)**	61.9 ±; 14.4	59 ±; 14.8	62.9 ±; 14.1
**SMI (kg/m^2^)**	9.96 ±; 1.7	11.8 ±; 1.8	9.3 ±; 1.1

BMI, body mass index; EW, excess weight; WC, waist circumference; HC, hip circumference; R, resistance; FFM, free fat mass; FM, fat mass; SMI, skeletal muscle index.

All participants gave written informed consent. The study was approved by the local ethics committee (protocol no. 21539). The data of all participants were entered into a registry and included anthropometric, clinical, and biological information and body composition determination. This study was performed in accordance with the ethical standards laid down in the 1964 Declaration of Helsinki and its later amendments.

### Anthropometry

Height was measured in meters with a stadiometer, body weight was measured in kilograms using a steelyard balance, and BMI was calculated as weight (in kilograms) divided by squared height (in meters). The waist circumference (WC, in centimeters) was measured with a tape placed parallel to the ground, at the midpoint between the upper margin of the iliac crest and the lower costal margin, considered at the level of the mid-axillary line.

Excess weight (EW) was calculated using the formula: [actual body weight − adjusted body weight]. The adjusted body weight was obtained with the formula: [ideal body weight + 0.4 (actual body weight − ideal body weight). For men, the ideal body weight is 50 kg + 2.3 kg for each inch over 5 ft, while that for women is 45.5 kg + 2.3 kg for each inch over 5 ft (13). Body composition was evaluated by BIA using the software Bodygram Plus Akern in order to estimate fat mass (FM) and free fat mass (FFM). In each BIA report test, the hydration level was reported. When the hydration level did not allow the test to be reliable, the patients were excluded from the evaluation and were not included in the sample.

The SMM was calculated using the equation by Janssen et al. (9):


$$\text{SM }\left(\text{kg}\right)=\left[\left(h_{2}/\text{BIA resistance}\times 0.401\right)+\left(\text{gender}\times 3.825\right)-\left(\text{age}\times 0.071\right)\right]+5.102$$

where height (*h*) is in centimeters and BIA resistance is in ohms. With regard to gender, M = 1 and F = 0. Age is in years. This equation has been developed and cross-validated by means of magnetic resonance measurements of whole-body SMM on a sample of 269 subjects with wide age (18–86 years) and BMI (16–48 kg/m^2^) ranges (14).

The SMI was calculated as the SMM normalized for squared height (15):


$$\text{SMI}=\text{SM}\left(\text{kg}\right)/h{\left(\text{m}\right)}^{2}.$$

### Statistical analysis

The patient characteristics were described as absolute frequencies and percentages for the categorical variables and as the mean ±; standard deviations (SD) for quantitative variables. Student’s *t*-test or the Mann–Whitney test was used to evaluate the differences in the quantitative variables according to normal distribution as evaluated using the Kolmogorov–Smirnov test. The *χ* ^2^ test was used to examine the association between qualitative variables.

### Cluster analysis and cutoff definition

An unsupervised *K*-means cluster with the number of clusters equal to 2 was performed (16). The SMI, height, weight, BMI, FFM, FM, EW, WC, hip circumference (HP), and resistance (*R*) were used as variables to assess the cluster analysis. The two obtained clusters were evaluated by clinicians: one reproduced the prototype of a sarcopenic patient and the other did not. Cluster membership was used as a proxy to distinguish sarcopenic from non-sarcopenic patients so that a receiver operating characteristic (ROC) curve could be estimated and a cutoff defined. Cutoffs with sensitivity and specificity of 90% were selected to define three ranges of patients: those with a high probability to be sarcopenic, those with a low probability to be sarcopenic, and those with an intermediate range of uncertainty. For the comparison between the patients defined as “sarcopenic,” “indeterminate,” and “non-sarcopenic,” ANOVA or the Kruskal–Wallis test with the Tukey procedure was used, or the Dunn test as *post hoc*. These tests allowed evaluating the differences and the characteristics between the three groups. For the selection of the sample to be used for the validation of the cutoffs, a matching procedure based on the nearest neighbor propensity score was carried out to limit the effect of confounding factors. Comparison of the anthropometric variables and the SMIs between the “training” and the “validation” group was carried out using Student’s *t*-test for continuous variables and the *χ* ^2^ test for nominal variables.

Analyses were conducted with R version 4.0.0. A *p*-value<0.05 was considered statistically significant.

For all statistical tests, *post-hoc* analysis was performed to define statistical power.

*Post-hoc* power analyses were carried out with G*Power version 3.1. The significance level considered was 0.05. The effect size was estimated based on the observed values. The test used was the unpaired Student’s *t*-test (to compare the quantitative variables between the sarcopenic, indeterminate, and non-sarcopenic groups). Based on this information, a power of at least 90% was obtained for each test, and even when the sample was smaller (78 men), the observed mean differences were very wide so that the test maintains a high power.

## Results

### Assessment of the prevalence of sarcopenic obesity using one of the cutoffs from the literature

The SMI cutoffs reported by De Rosa et al. (17) were used. These cutoffs were identified in a group of 500 young, normal weight adults from Southern Italy (400 women and 100 men) using the 1 SD method. The gender-specific cutoffs were 7.3 and 9.5 kg/h^2^ for women and men, respectively. Using these cutoffs, the observed prevalence of SO in our cohort of patients with severe obesity was 2.3%.

### Identification of new cutoffs and the prevalence of sarcopenia in patients with severe obesity (“training group”)

As a first method, the mean of the SMI values in the study population was calculated by stratifying the patients based on gender. Participants with a SMI 1 standard deviation lower than the gender-specific mean were defined as sarcopenic. The means of the SMI values were 9.3 ±; 1.1 kg/h^2^ for women and 11.7 ±; 1.5 kg/h^2^ for men. The gender-specific cutoffs were 8.2 and 10.2 kg/h^2^ for women and men, respectively.

**Figure 1:**
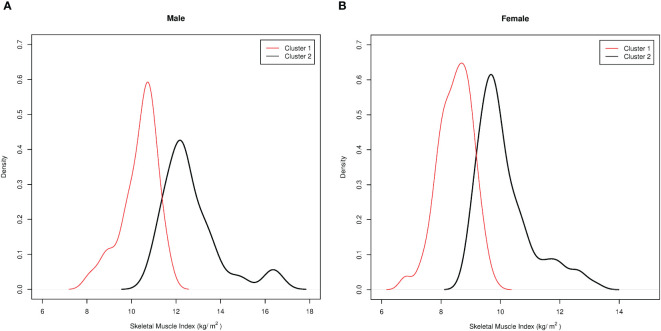
Pathological and non-pathological clusters based on the skeletal muscle index (SMI) values according to the indirect approach, “unsupervised *K*-means cluster analysis.” Cluster membership was used as a proxy to distinguish the sarcopenic from the non-sarcopenic patients. **(A)** Pathological/sarcopenic (*red*) and non-pathological/non-sarcopenic (*black*) clusters in men. **(B)** Pathological/sarcopenic (*red*) and non-pathological/non-sarcopenic (*black*) clusters in women.

**Figure 2:**
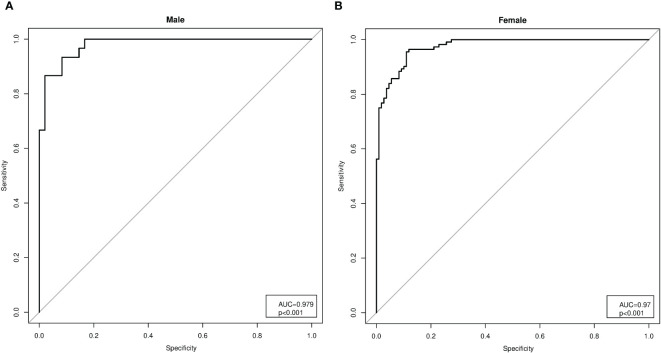
Analysis of the receiver operating characteristic (ROC) curves for the gender-specific cutoffs of the skeletal muscle index (SMI). **(A)** Analysis of the ROC curves in men (AUC = 0.979, *p*< 0.001), with SMI cutoff of 11.3 kg/h^2^ (specificity of 91% and sensitivity of 93%). **(B)** Analysis of the ROC curves in women (AUC = 0.979, *p*< 0.001), with SMI cutoff of 9.2 kg/h^2^ (sensitivity and specificity of 94%).

As a second method, an indirect approach referred to as *K*-means unsupervised cluster was used. The SMI, height, weight, BMI, FFM, FM, EW, WC, HP, and *R* were used as variables to identify two different clusters defined as pathological and non-pathological on the basis of the SMI values (**Figure 1**). The anthropometric features and body composition of the patients in the two clusters stratified by gender are shown in **Table 2**. Two gender-specific cutoffs of SMI from the ROC curves were identified (**Figure 2**): SMI of 9.2 kg/h^2^ for women (with sensitivity and specificity of 94%) and SMI of 11.3 kg/h^2^ for men (with a specificity of 91% and a sensitivity of 93%). The gender-specific cutoff points and the respective prevalence of SO are shown in **Table 3**. The prevalence rates of sarcopenia were 14.7% (44/300) and 47.6% (143/300) with the standard deviation method and the cluster method, respectively.

**Table 2:** Anthropometric features and body composition of the two clusters stratified by gender.

Parameters	Women (222, 74%)			Men (78, 26%)		
	Pathological Mean ±; SD	Non-pathological Mean ±; SD	*p*	Pathological Mean ±; SD	Non-pathological Mean ±; SD	*p*
**Weight (kg)**	114.5 ±; 5.62	128.7 ±; 18.2	<0.0001	129.9 ±; 17.6	150.2 ±; 21.7	0.0001
**BMI (kg/m^2^)**	44.1 ±; 5.9	49.2 ±; 6.2	<0.0001	43.8 ±; 5.2	48.9 ±; 6.2	0.0003
**EW (kg)**	36.5 ±; 8.4	44.9 ±; 9.9	<0.0001	36.7 ± 9.7	48 ±; 11.3	<0.0001
**WC (cm)**	120.3 ±; 12.2	130.5 ±; 14.8	<0.0001	132.5 ±; 10.1	141.4 ±; 11.3	0.003
**HC (cm)**	131.5 ±; 11.1	137.2 ±; 12.8	0.0003	127.7 ±; 12.3	132.7 ±; 10.2	0.07
**R (Ohm)**	527.3 ±; 37.8	431.3 ±; 37.4	<0.0001	483 ±; 49.9	377.9 ±; 37.3	<0.0001
**FFM (kg)**	51.4 ±; 6.06	58.4 ±; 8.4	<0.0001	64.4 ±; 10.5	82.7 ±; 14.4	<0.0001
**FM (kg)**	59.3 ±; 12.9	66.8 ±; 14.9	0.0003	57.1 ±; 13.3	60.3 ±; 15.7	0.3
**SMI (kg/m^2^)**	8.48 ±; 0.57	10.12 ±; 0.9	<0.0001	10.3 ±; 0.8	12.6 ±; 1.3	<0.0001

BMI, body mass index; EW, excess weight; WC, waist circumference; HC, hip circumference; R, resistance; FFM, free fat mass; FM, fat mass; SMI, skeletal muscle index.

**Table 3:** Prevalence of sarcopenic obesity in the whole cohort according to different cutoffs.

Parameters	De Rosa et al. (17)			Mean −1 SD^a^			Cluster^b^		
	F	M	Total	F	M	Total	F	M	Total
**SMI cutoff (kg/m^2^)**	7.32	9.53		8.2	10.2		9.2	11.3	
**Sarcopenic obesity prevalence, *n* (%)**	3 (1.3)	4 (5.1)	7 (2.3)	35 (15.8)	9 (11.5)	44 (14.7)	111 (50)	32 (41)	143 (47.6)

SMI, skeletal muscle index; F, females; M, males.

a Mean −1SD of the SMI values in our population.

b K-means unsupervised cluster.

### Stratification of the study population according to the two identified cutoff points

**Figure 3:**
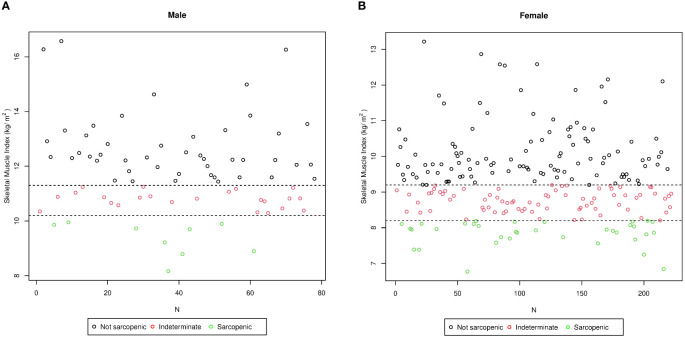
Stratification of the study population (training group) into three subgroups according to the skeletal muscle index (SMI) values in men **(A)** and in women **(B)**. *N*, number of patients. The sarcopenic obese group (*green circle*) included subjects affected by sarcopenia based on both cutoffs (SMIs of<8.2 and<10.2 kg/h^2^ for women and men, respectively, *n* = 44); the non-sarcopenic group (*black circle*) included subjects classified as not affected by sarcopenia using both cutoffs (SMIs of >9.2 and >11.3 kg/h^2^ for women and men, respectively, *n* = 157); and the intermediate group (*red circle*) included subjects classified as sarcopenic by the cluster method and as non-sarcopenic by the standard deviation method (8.2< SMI< 9.2 kg/h^2^ for women and 10.2< SMI< 11.3 kg/h^2^ for men, *n* = 99).

The study population (the training group) was stratified into three subgroups of patients (**Figure 3**): 1) non-sarcopenic (NS) patients for both identified cutoffs (SMIs of >9.2 and >11.3 kg/h^2^ for women and men, respectively, *n* = 157); 2) sarcopenic (S) patients for both cutoffs (SMIs of<8.2 and<10.2 kg/h^2^ for women and men, respectively, *n* = 44); and 3) indeterminate (I) patients (8.2< SMI<9.2 kg/h^2^ for women and 10.2< SMI<11.3 kg/h^2^ for men, *n* = 99) who were sarcopenic when using the cluster method but non-sarcopenic when using the standard deviation method. To verify whether the I patients were similar to the S or NS patients, the three groups were compared. As shown in **Table 4**, in indeterminate patients, the lean mass was similar to that observed in the sarcopenic group (55.77 ±; 10.62 kg *vs*. 53.03 ±; 7.14 kg, *p* = 0.13). In contrast, a lower lean mass was found in the indeterminate group when compared with the non-sarcopenic group (55.77 ±; 10.62 *vs*. 65.36 ±; 15.37 kg, *p*< 0.0001).

**Table 4:** Comparison between the sarcopenic (S), indeterminate (I), and non-sarcopenic (NS) patients for the anthropometric parameters and body composition defined by bioelectrical impedance analysis (BIA).

Baseline	NS (157, 52.3%) Mean ±; SD	I (99, 33%) Mean ±; SD	S (44, 14.7%) Mean ±; SD	*p*
Age (years)	42.08 ±; 9.01	43.67 ±; 9.12	42.67 ±; 9.26	0.3
Weight (kg)	133.97 ±; 21.65	121.44 ±; 19.61	114.62 ±; 16.27	<0.0001^a,b^
BMI (kg/m^2^)	48.93 ±; 6.43	45.75 ±; 5.83	41.18 ±; 4.37	<0.0001^a,b,c^
EW (kg)	45.31 ±; 10.67	39.04 ±; 9.39	33.26 ±; 7.08	<0.0001^a,b,c^
WC (cm)	132.85 ±; 15	126.13 ±; 13.32	119.87 ±; 12.55	<0.0001^a,b,c^
HC (cm)	135.66 ±; 13.45	132.48 ±; 12.63	126.91 ±; 9.26	0.001^a,c^
FM (kg)	64.22 ±; 15.47	60.73 ±; 14.57	57.1 ±; 9.94	0.01^a^
FFM (kg)	65.36 ±; 15.37	55.77 ±; 10.62	53.03 ±; 7.14	<0.0001^a,b^

BMI, body mass index; EW, excess weight; WC, waist circumference; HC, hip circumference; FM, fat mass; FFM, free fat mass.

a Significant difference between NS and S patients.

b Significant difference between NS and I patients.

c Significant difference between I and S patients.

Since the patients in the indeterminate group were similar to those included in the sarcopenic group and were classified as sarcopenic by the cluster method, we speculated that this might be the best cutoff to identify sarcopenia in patients with severe obesity.

### Validation of the cutoff points obtained with the cluster method

To validate the cutoffs identified using the cluster method, a second sample consisting of 300 patients (the “validation group”) with severe obesity and were candidates to bariatric surgery (mean BMI = 44.9 ±; 6.7 kg/m^2^) was used. The characteristics of the patients in the validation group and the comparison with the training group are shown in **Table 5**. Although the patients had different anthropometric and body composition characteristics, similar rates of sarcopenia in the patients in both groups (56% in the validation group and 47.7% in the training group) were observed.

**Table 5:** Comparison between the training and validation groups for the anthropometric and clinical parameters and body composition defined by bioelectrical impedance analysis (BIA).

Parameter	Training (*N* = 300) Mean ±; SD	Validation (*N* = 300) Mean ±; SD	*p*
Men, *n* (%)	78 (26)	79 (26.3)	1
Age (years)	42.7 ±; 9.08	47.5 ±; 9.6	<0.001
BMI (kg/m^2^)	46.7 ±; 6.5	44.9 ±; 6.7	0.001
WC (cm)	128.7 ± 14.9	122.1 ±; 14.2	<0.001
HC (cm)	133.3 ±; 13	130.9 ±; 13	0.03
FM (kg)	62 ±; 14.7	56.5 ±; 13.6	<0.001
FFM (kg)	60.4 ±; 14	63.9 ±; 14.4	0.002
R (Ohm)	463.1 ±; 67.8	462.8 ±; 72.6	0.9
SMI (kg/m^2^)	9.95 ±; 1.66	9.62 ±; 1.5	0.007
Sarcopenic, *n* (%)	143 (47.7)	170 (56)	0.03

BMI, body mass index; WC, waist circumference; HC, hip circumference; FM, fat mass; FFM, free fat mass; R, resistance; SMI, skeletal muscle index.

A sub-analysis through a matching procedure (using only BMI and age as the variables) based on the “propensity scores” between the validation group and the training group (consisting of a subgroup of 215 patients selected from the starting cohort) was also performed. After matching, the rates of sarcopenia were similar in the two groups of patients (50.2% in the validation group and 53% in the training group, *p* = 0.6) (**Table 6**).

**Table 6:** Comparison between the training and validation groups for the anthropometric and clinical parameters and body composition defined by bioelectrical impedance analysis (BIA) after a matching procedure for age and body mass index (BMI).

Parameter	Training (*n* = 215) Mean ±; SD	Validation (*n* = 215) Mean ±; SD	*p*
Men, *n* (%)	54 (25.1)	54 (25.1)	1
Age (years)	44.3 ±; 8.28	45.1 ±; 9.17	0.3
BMI (kg/m^2^)	45.9 ±; 6	45.4 ±; 6.92	0.3
WC (cm)	127.2 ± 14.75	122.1 ±; 14.4	<0.001
HC (cm)	132.4 ±; 12.6	131.4 ±; 13.7	0.4
FM (kg)	60.6 ±; 13.55	57.03 ±; 14.06	0.008
FFM (kg)	61 ±; 13.9	64.5 ±; 15.15	0.01
R (Ohm)	461.4 ±; 69	458.3 ±; 71.3	0.6
SMI (kg/m^2^)	9.94 ±; 1.7	9.75 ±; 1.5	0.2
Sarcopenic, *n* (%)	108 (50.2)	114 (53)	0.6

BMI, body mass index; WC, waist circumference; HC, hip circumference; FM, fat mass; FFM, free fat mass; R, resistance; SMI, skeletal muscle index.

## Discussion

SO is a clinical–functional condition characterized by an alteration of the body composition in which there is an excess of FM and a deficit of muscle mass (10). A recent systematic literature review has shown heterogeneity of the data in terms of the diagnostic criteria and the methodological tools used (12). ESPEN and EASO have recently published a consensus to establish a definition and univocal diagnostic criteria (10): the diagnosis is performed in two steps: firstly with the assessment of the skeletal muscle functional parameters and subsequently with the assessment of the body composition using DXA, BIA, or, when possible, CT/MRI (10). MRI is considered the gold standard as it is also able to evaluate intramuscular fat (8); however, BIA is the most used tool in clinical practice for patients with obesity due to its low cost, wide availability, and portability (3).

With regard to the various indices of sarcopenia assessed using BIA, the SMI is widely described in the literature and is the only parameter that appears to be predictive of functional impotence and disability (14, 15). Nevertheless, there is currently no univocal cutoff for SMI assessed by BIA that is valid for the definition of sarcopenia in patients with morbid obesity (10).

The recent consensus recommends using the SMM as the skeletal muscle normalized for weight to evaluate SO (10). However, there could be a risk of overestimating the presence of sarcopenia, as also already highlighted by De Rosa et al. (17). The EWGSOP2 recommends the use of cutoffs placed to measure 1 and 2 standard deviations below the mean SMI values of a young reference population for the definition of moderate and severe sarcopenia, respectively (7). Nevertheless, in several studies, it has been observed that the SMI is significantly higher in subjects with obesity than that in subjects with normal weight (17, 18). Thus, a SMI with normal cutoffs has the risk of underestimating the presence of sarcopenia because, in patients with obesity, both absolute fat and muscle mass increase. Therefore, the identification of new SMI cutoffs could be considered for the correct evaluation of this condition.

Indeed, in the study population, by applying the SMI cutoffs calculated in a cohort of healthy subject from a region in Southern Italy who are similar in race, environment, and geographical area to those in our population (17), a very low rate of sarcopenia was found (2.3%). Conversely, when the SMI cutoffs were obtained from a group of subjects affected by morbid obesity, the rate of sarcopenia rose from 2.7% to 14.7% when using standard deviation and from 2.7% to 47.6% when using the cluster method, confirming that the SMI cutoffs validated in the healthy population might not be adequate for patients with obesity.

Furthermore, also by using the SMI cutoffs identified in the cohort of patients with severe obesity, the rate of sarcopenia observed was significantly different and was dependent on the method used. Nevertheless, additional analysis demonstrated that the cluster method is probably more suitable for the purpose of correctly identifying the presence of sarcopenia. To validate these cutoff points, the prevalence of sarcopenia in a second sample consisting of 300 patients affected by morbid obesity (the validation group) was calculated. A high rate of sarcopenia (56%) was also observed in this group, albeit statistically different from the sarcopenia rate in the training group. However, after matching the patients of the two groups by BMI and age, the rates of sarcopenia became similar in the two patient groups, thus confirming the hypothesis that the cutoffs obtained by the cluster method can be adapted well to patients with severe obesity.

To date, most of the data reported in the literature on SO are those of elderly people with heterogeneous BMI values (19–22). Only a few studies have dealt with the prevalence of SO in the adult population (17, 18, 23). As a result of the new cutoffs calculated using cluster analysis, the study reports a higher risk of sarcopenia, also at an earlier age (mean age = 43 years), which calls into question whether SO is not just a geriatric syndrome.

Moreover, sarcopenic patients had significantly lower weight, BMI, EW, WC, and HC values. In patients with obesity, the increase in body mass is due to the increase in both fat and lean mass. The relative contribution of these two components to EW is partly influenced by age and by the proportion of FM and lean mass in these subjects before weight gain (24). Therefore, in the study population, the higher BMI values in non-sarcopenic patients could be due to the greater components of lean mass and FM.

The present study has strengths and limitations. One of its limitations is the use of BIA as a tool to assess body composition, which is still an indirect measurement technique based on algorithms that, as much as have been elaborated, do not represent a direct system of assessment. BIA, despite being practical to use and having wide availability, is influenced by the patient’s hydration status (7).

In addition, any functional test assessing muscle strength, which is considered a necessary component in the evaluation of the presence/absence of sarcopenia, was not performed, and it is one of the first steps of the SO diagnostic algorithm. An assessment of the dietary intake and physical activity was not performed either.

This study also has certain strengths. It included a large cohort of adults with severe obesity of both genders in a real-world clinical setting. In addition, to our knowledge, this is the first study that attempted to identify specific SMI cutoffs in patients with severe obesity. These new cutoffs could be considered robust as they were calculated from a large group of patients with morbid obesity (*n* = 300) from the same country with the same disease and were validated in a similar cohort of patients. Finally, another strength of the cutoffs determined in this study is their potential utility in clinical practice, being simple and easily applicable.

## Conclusions

The study showed that the current cutoffs used for SO diagnosis in the general population are not suitable for patients with morbid obesity. The development of new cutoffs, calculated based on severely obese patients with the same disease might be better adapted. However, before these new criteria can be implemented in clinical routine, they need to be validated in other patient cohorts with severe obesity and verified whether they are correlated with muscle strength and physical disabilities.

## Data availability statement

The raw data supporting the conclusions of this article will be made available by the authors, without undue reservation.

## Ethics statement

The study was conducted in accordance with the Declaration of Helsinki, and approved by Siena Ethics Committee “Office of Ethical Affairs” (protocol code 21539, date approval 21/01/2022). The studies were conducted in accordance with the local legislation and institutional requirements. The participants provided their written informed consent to participate in this study.

## Author contributions

ABu: Writing – original draft, Writing – review & editing. AC: Writing – original draft, Writing – review & editing. NB: Writing – original draft, Writing – review & editing. CC: Writing – original draft, Writing – review & editing. MS: Writing – original draft, Writing – review & editing. ABo: Writing – original draft, Writing – review & editing. GI: Writing – original draft, Writing – review & editing. IS: Writing – original draft, Writing – review & editing. AT: Writing – original draft, Writing – review & editing. CV: Writing – original draft, Writing – review & editing. GV: Writing – original draft, Writing – review & editing. MC: Writing – original draft, Writing – review & editing.
